# A glycolysis-related gene signatures in diffuse large B-Cell lymphoma predicts prognosis and tumor immune microenvironment

**DOI:** 10.3389/fcell.2023.1070777

**Published:** 2023-01-23

**Authors:** Yingying Cui, Changsen Leng

**Affiliations:** ^1^ State Key Laboratory of Oncology in South China, Collaborative Innovation Center for Cancer Medicine, Sun Yat-sen University Cancer Center, Guangzhou, China; ^2^ Department of Hematologic Oncology, Sun Yat-sen University Cancer Center, Guangzhou, China; ^3^ Department of Thoracic Surgery, Sun Yat-sen University Cancer Center, Guangzhou, China; ^4^ Guangdong Esophageal Cancer Institute, Guangzhou, China

**Keywords:** diffuse large B-cell lymphoma, prognostic model, glycolysis, tumor microenvironment, immunotherapy

## Abstract

**Background:** Diffuse large B-cell lymphoma (DLBCL) is the most common type of lymphoma which that highly aggressive and heterogeneous. Glycolysis has been implicated in the regulation of tumor microenvironment (TME) and development. In this study, we aimed to establish a glycolysis-related prognostic model for the risk stratification, prognosis prediction, and immune landscape evaluation in patients with DLBCL.

**Methods:** Three independent datasets GSE181063, GSE10846, and GSE53786 containing gene expression profiles and clinical data were downloaded from the Gene Expression Omnibus (GEO) database. The glycolysis-related prognostic model was developed with Cox and Least Absolute Shrinkage and Selector Operation (LASSO) regression and validated. A nomogram integrating clinical factors and glycolytic risk scores was constructed. The composition of the TME was analyzed with the ESTIMATE algorithm and single-sample gene set enrichment analysis (ssGSEA).

**Results:** A glycolytic risk model containing eight genes was developed. The area under the receiver operating characteristic (ROC) curve (AUC) for the 1-, 3-, and 5-year was 0.718, 0.695, and 0.688, respectively. Patients in the high-risk group had significantly lower immune scores, elevated tumor purity, and poorer survival compared with those in the low-risk group. The nomogram constructed based on glycolytic risk score, age, Eastern Cooperative Oncology Group performance status (ECOG-PS), use of rituximab, and cell of origin (COO) displayed better prediction performance compared with the International Prognostic Index (IPI) in DLBCL. The glycolytic risk score was negatively correlated with the infiltration level of activated CD8 T cells, activated dendritic cells, natural killer cells, and macrophages and immune checkpoint molecules including *PD-L2*, *CTLA4*, *TIM-3*, *TIGIT*, and *B7-H3*.

**Conclusion:** These results suggested that the glycolytic risk model could accurately and stably predict the prognosis of patients with DLBCL and might unearth the possible explanation for the glycolysis-related poor prognosis.

## 1 Introduction

Diffuse large B-cell lymphoma (DLBCL) is the most common histological subtype of lymphoma worldwide, accounting for approximately 25%–30% of all non-Hodgkin lymphoma (Sehn and Salles, 2021). Currently, RCHOP (rituximab, cyclophosphamide, adriamycin, vincristine, and prednisone) regimen is considered the standard first-line chemotherapy (Coiffier et al., 2010). The prognosis of DLBCL patients is often prospectively predicted using the International Prognostic Index (IPI) based on the clinical features before treatment (International Non-Hodgkin’s Lymphoma Prognostic Factors, P, 1993). However, approximately 40% of patients with DLBCL have a poor response to treatment and develop relapsed/refractory disease after first-line therapy (Coiffier et al., 2010). Furthermore, even patients with DLBCL in the same IPI risk category exhibit different clinical outcomes owing to the high tumor heterogeneity (Ruppert et al., 2020). Hence, there is an urgent need to explore novel molecular biomarkers for accurate prediction of prognosis and reveal ideas for developing specific therapeutic targets.

Metabolic reprogramming is one of the most important features of malignant tumors. By altering metabolism and energy production ways, neoplastic cells satisfy the needs of sustainable proliferation, invasion, and metastasis (Faubert et al., 2020). Aerobic glycolysis is among the key features of metabolic reprogramming and is common among cancer cells. In 1924, Otto Heinrich Warburg first demonstrated that tumor cells preferentially converted glucose into lactate *via* glycolysis even with sufficient oxygen, which differed from normal cells in which glucose is metabolized *via* oxidative phosphorylation in the mitochondria. This phenomenon is referred to Warburg effect (DeBerardinis and Chandel, 2020). The glucose will not be metabolized to lactate through glycolysis in normal tissues when oxygen is available. Normal tissues convert glucose into lactic acid only under oxygen deprivation. By contrast, tumor tissues metabolize and convert about 66% of ingested glucose to lactate even in the presence of oxygen (Kalyanaraman, 2017; San-Millan and Brooks, 2017). Therefore, through glycolysis, lactate levels in cancer tissue are remarkably increased by 40-fold and are highly related to cancer aggressiveness and poor prognosis. By upregulating VEGF and TGF-β2 expression, inhibiting monocyte migration, suppressing T-cell activation, and promoting the release of IL-6, glycolysis promotes angiogenesis, cell migration, cell metastasis, and immune escape in the tumor microenvironment (San-Millan and Brooks, 2017). Glycolysis contributes about 50%–60% of the total ATP in cancer cells at most (Zheng, 2012). Although glycolysis is less efficient in producing ATP compared to mitochondrial oxidative phosphorylation (OXPHOS), the rate of ATP generation by glycolysis is approximately 100 times faster than that of OXPHOS, which sufficiently meets the energy demand of the rapid tumor proliferation (Ganapathy-Kanniappan and Geschwind, 2013). This demonstrates the advantages of glycolysis over OXPHOS in cancer metabolism. Besides, accumulating evidence has revealed that the tumor immune microenvironment (TIME) influences the efficacy of immunotherapy and prognosis of cancer patients (Karube, 2021; Watanabe, 2021). Apart from the metabolic function, glycolysis-induced hypoxia and lactate accumulation affect the TIME and the function of immune cells. It regulates the antitumor immune response by suppressing lymphocyte proliferation and cytotoxic activity, thereby indirectly promoting cancer progression (Wu et al., 2020). For example, metabolites of glycolysis were reported to attenuate the infiltration level of CD8^+^ T cells and natural killer cells and facilitate infiltration of immunosuppressive T cells.

By providing energy and synthesizing key molecules and enzymes, glycolysis actively regulates the proliferation, metastasis, gene expression regulation, and biosynthesis processes in cancer cells (Faubert et al., 2020). Oslund et al. found that one of the biological functions of bisphosphoglycerate mutase (BPGM) is to regulate serine synthesis by controlling the levels of intermediate glycolytic products (Oslund et al., 2017). Upregulated BPGM has been associated with the poor prognosis of hepatocellular carcinoma patients (Cai et al., 2020). Peptidylglycine alpha-amidating monooxygenase (PAM), a copper- and ascorbate-dependent enzyme, coordinates tissue-specific responses to hypoxia (Rao et al., 2021). Increased expression of PAM has been proven to be an independent predictor of favorable prognosis in patients with neuroendocrine neoplasms (Horton et al., 2020). However, there are no reliable glycolytic biomarkers for predicting the prognosis of DLBCL patients.

In this study, we developed a glycolysis-related prognostic gene signature in DLBCL patients and validated its prediction performance in different cohorts. We then comprehensively analyzed the TIME and immune cell infiltration at different risk levels. This study presents an effective prognostic model and reveals new therapeutic targets for DLBCL that are expected to promote individualized treatment of patients.

## 2 Methods

### 2.1 Data acquisition and preprocessing

The gene expression profile and clinicopathological characteristics of patients with DLBCL of GSE181063 (*n* = 1310), GSE10846 (*n* = 420), and GSE53786 (*n* = 119) datasets were downloaded from Gene Expression Omnibus (GEO, https://www.ncbi.nlm.nih.gov/geo/). The GSE181063 profile was used as the training cohort and based on the Illumina HumanHT-12 WG-DASL V4.0 R2 expression beadchip platform (Illumina, Bethesda, MD, United States). The independent external validation cohorts GSE10846 and GSE53786 were based on the Affymetrix Human Genome U133 Plus 2.0 Array platform (Affymetrix, Santa Clara, CA, United States). The inclusion criteria of DLBCL patients were as follows: 1) histologically confirmed DLBCL; 2) had detailed corresponding clinicopathological information; 3) initially treated with RCHOP or CHOP regimen; 4) overall survival (OS) time ≥30 days. The study was conducted in accordance with the Declaration of Helsinki (as revised in 2013).

Glycolysis-related gene sets (REACTOME_GLYCOLYSIS, KEGG_GLYCOLYSIS_GLUCONEOGENESIS, HALLMARK_GLYCOLYSIS, BIOCARTA_GLYCOLYSIS_PATHWAY, BIOCARTA_FEEDER_PATHWAY, GOBP_POSITIVE_REGULATION_OF_GLYCOLYTIC_PROCESS, GOBP_NEGATIVE_REGULATION_OF_GLYCOLYTIC_PROCESS, GOBP_GLYCOLYTIC_PROCESS_THROUGH_FRUCTOSE_6_PHOSPHATE) were obtained from the Molecular Signatures Database (MSigDB v7.5.1, http://www.broad.mit.edu/gsea/msigdb/).

### 2.2 Glycolytic risk model construction and validation

Cox and least absolute shrinkage and selection operator (LASSO) regression analyses were used in the training cohort to identify the glycolysis-related prognostic model. Firstly, we performed the univariate Cox regression analysis with the “survival” package in R to identify survival-associated glycolytic genes. Genes with *p* < 0.01 were selected. Then, LASSO regression was carried out to further select key glycolytic genes by using the “glmnet” package in R. Finally, the multivariate Cox analysis was performed to establish the glycolytic risk model. The risk model was constructed by each glycolytic gene expression value (G) and the corresponding coefficient (β). The $risk\;score=\overset{j}{\underset{n=1}{\sum }}\left(\beta j*Gj\right)$ . Patients with DLBCL were classified into low- and high-risk groups according to the median value of the risk score. Heatmap of the risk gene expression, risk score distribution plots, and survival status scatter plots of the low- and high-risk groups were established to assess the risk model. The predictive accuracy of the risk model was further evaluated by Kaplan-Meier (K-M) analyses and time-dependent receiver operating characteristic (ROC) curves by using the “survival”, “survminer”, and “timeROC” packages in R.

GSE181063 dataset was selected as the training cohort. The other two independent datasets, GSE510846 and GSE53786 were used as external validation cohorts. Additionally, samples in the GSE181063 dataset were randomly divided into two internal validation cohorts at a 1:1 ratio by using the “caret” package in R. Clinical features of the training and validation cohorts are shown in Table 1. The comparison of clinical characteristics between two internal validation cohorts is summarized in Supplementary Table S1. The performance of the risk model was assessed in all validation cohorts. *p* < 0.05 was considered as the significance threshold.

**TABLE 1:** Clinicopathological characteristics of patients with DLBCL enrolled from the GEO database.

	GSE181063	GSE10846	GSE53786
	(*n* = 559)	(*n* = 305)	(*n* = 83)
Age (year), No. (%)			
≤60	184 (32.9%)	146 (47.9%)	38 (45.8%)
>60	375 (67.1%)	159 (52.1%)	45 (54.2%)
Gender, No. (%)			
Male	305 (54.6%)	171 (56.1%)	51 (61.4%)
Female	254 (45.4%)	134 (43.9%)	32 (38.6%)
ECOG-PS, No. (%)			
<2	476 (85.2%)	230 (75.4%)	57 (68.7%)
≥2	83 (14.8%)	75 (24.6%)	26 (31.3%)
LDH, No. (%)			
Normal	214 (38.3%)	157 (51.5%)	43 (51.8%)
Elevated	345 (61.7%)	148 (48.5%)	40 (48.2%)
AnnAnbor stage, No. (%)			
I–II	221 (39.5%)	144 (47.2%)	36 (43.4%)
III–IV	338 (60.5%)	161 (52.8%)	47 (56.6%)
COO, No. (%)			
GCB	265 (47.4%)	133 (43.6%)	31 (37.3%)
nonGCB	294 (52.6%)	172 (56.4%)	52 (62.7%)
Rituximab, No. (%)			
Yes	549 (98.2%)	163 (53.4%)	49 (59.0%)
No	10 (1.8%)	142 (46.6%)	34 (41.0%)
Extranodal sites, No. (%)			
<2	462 (82.6%)	282 (92.5%)	77 (92.8%)
≥2	97 (17.4%)	23 (7.5%)	6 (7.2%)

DLBCL, Diffuse large B-cell lymphoma; GEO, Gene expression omnibus; No, Number; ECOG-PS, Eastern cooperative oncology group performance status; LDH, Lactate dehydrogenase; COO, Cell of origin; GCB, Germinal center B cell.

### 2.3 Association between the clinical features and glycolytic risk model

To explore whether the risk model could accurately predict prognosis in the subgroup of clinical parameters including age, gender, Ann Arbor stage, ECOG-PS, lactate dehydrogenase (LDH) level, COO (cell of origin), number of extranodal sites, and B symptoms, we compared the survival between different risk groups based on the stratification of clinical parameters. To further figure out the relationship between the risk model and clinical factors, we compared the risk score distribution in subgroups of the clinical parameters by using the “ggpubr” package in R.

### 2.4 Independent prognostic factors and nomogram construction

Univariate and multivariate Cox regression analyses were applied in the training and validation (GSE10846) cohorts to determine whether the risk score and clinical parameters were independent prognostic factors in DLBCL patients. Next, we developed a prognostic nomogram to integrate independent prognostic factors with “rms”, “foreign”, and “survival” packages in R. According to the risk contribution to OS, prognostic factors were assigned score points. The total score points were calculated to predict the 1-, 3-, and 5-year prognosis of DLBCL patients. The Harrell’s concordance index (C-index) and calibration curves were performed to evaluate the consistency of the actual and predicted survival probabilities of patients by using the R packages “rms”, “foreign”, and “survival”. ROC curves were drawn and the area under the ROC curve (AUC) was calculated to assess the prognostic performance of nomogram and IPI by using the “survivalROC” package in R.

### 2.5 Tumor microenvironment (TME) and immune cell infiltration

TME consists of tumor cells, immune cells, stromal cells, and non-cellular components (Bader et al., 2020). Immune scores and stromal scores were calculated in each sample using the “estimate” package in R software (Yoshihara et al., 2013). They represented the proportion of stromal and immune cells in TME and were incorporated as ESTIMATE scores, to further infer the tumor purity in each DLBCL patient. The survival was compared between different score levels with a *t*.test.

To further explore immune cells (encompass activated CD8 T cells, activated CD4 T cells, activated B cells, activated dendritic cells, natural killer cells, and macrophages) infiltration profile in DLBCL patients, the single-sample gene set enrichment analysis (ssGSEA) was performed based on gene expressions of immune cell-specific markers to quantify the enrichment level of each immune cell type with “GSVA” package in R (Bindea et al., 2013). The correlation between the immune cells infiltration and risk model was evaluated with Pearson correlation coefficient and displayed by using the “ggplot2” package in R.

### 2.6 Association of the immune modulator and glycolytic risk model

Immune checkpoint inhibitors (ICIs) prevent checkpoint proteins from binding with the partner proteins and prompt T cells to kill cancer cells (Galluzzi et al., 2020). The immune checkpoint molecules, including PD-1, PD-L1, PD-L2, CTLA-4, LAG3, TIM-3, TIGIT, B7-H3, B7-H4, and CD47, were considered promising targets in B-cell lymphoma in previous studies (Ansell et al., 2009; Lee et al., 2017; Advani et al., 2018; Xu-Monette et al., 2018; Jiang et al., 2019; Hatic et al., 2021; Shimada et al., 2021; Lee et al., 2022). The association between immune checkpoint molecule expression and risk score were assessed with the Pearson correlation coefficient and presented using the “corrplot” package in R to explore the potential effect of our glycolytic risk model in tumor immunotherapy.

### 2.7 Measuring the glycolytic risk genes expression at the protein level

The immunohistochemical staining of the glycolytic risk genes in lymphoma patients and normal controls was obtained from the Human Protein Atlas (HPA) database (http://www.proteinatlas.org/) (Liu et al., 2022).

### 2.8 Analysis of the difference of metabolites

Details of DLBCL cell lines information were downloaded from Cancer Cell Line Encyclopedia (CCLE, https://portals.broadinstitute.org/ccle/data), including cell lines annotations, mRNA expression, and metabolites. The correlation analysis between gene expression and metabolites were performed. Metabolites with *p*-value < 0.05 and |*cor*| > 0.5 were selected in this study and shown in the heat map using “ggplot2” package in R software (Sui et al., 2022).

### 2.9 Evaluation of the sensitivity of chemotherapeutic agents

To predict the drug sensitivity based on the heterogenicity of tumor samples, the half-maximal inhibitory concentration (IC50) of DLBCL patients in high- and low-risk groups was estimated by using the “pRRophetic” package in R. The potential anti-cancer compounds were screened based on the drug response data in Genomics of Drug Sensitivity in Cancer (GDSC) database (Wang et al., 2021).

## 3 Gene set variation analysis (GSVA)

GSVA between high- and low-risk groups were performed by using the “GSVA package” in R to identify commonly activated/inhibited signaling pathways. The Hallmark gene set obtained from MSigDB was used as the reference gene set. The cut-off criteria were set as the *p*-value < 0.05 and the *t* value > 2 (Zhang et al., 2021).

### 3.1 Statistical analysis

All data analyses were performed in R version 4.0.3. *p* < 0.05 was considered statistically significant.

## 4 Results

### 4.1 Patient characteristics

The study included 947 eligible patients from the training cohort (GSE181063, *n* = 559) and two external validation cohorts (GSE10846, *n* = 305; GSE53786, *n* = 83) (Lenz et al., 2008; Scott et al., 2014; Lacy et al., 2020). Patient baseline characteristics are shown in Table 1. The training cohort was randomly allocated into two internal validation cohorts at a 1:1 ratio. There were no significant differences in clinical factors between the two groups (Chi-square test, *p* > 0.05). Results are shown in Supplementary Table S1.

### 4.2 Construction of the glycolytic risk model

**FIGURE 1:**
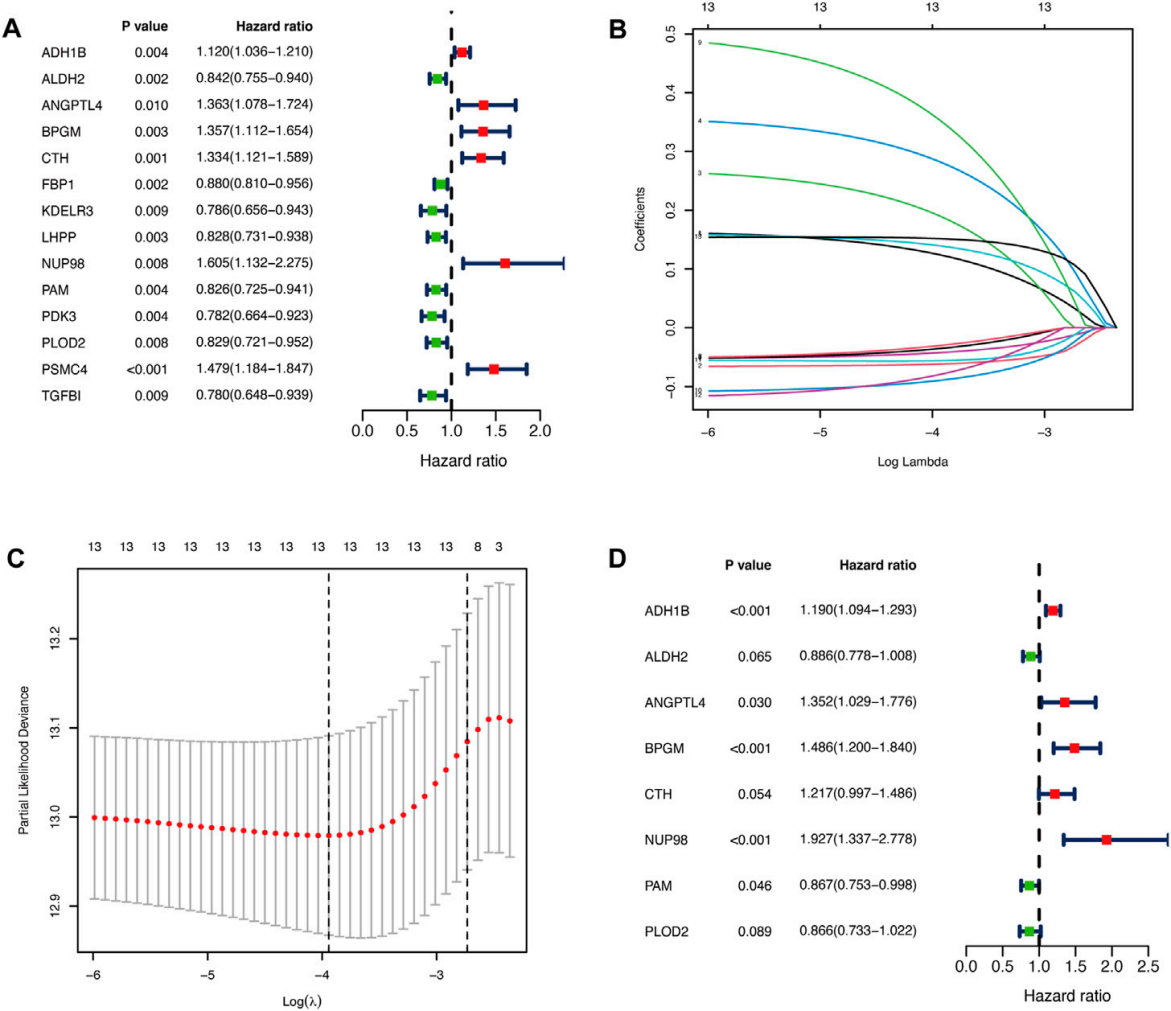
Identification of glycolysis-related prognostic genes in patients with DLBCL. **(A)**Fourteen glycolysis-related candidate genes were selected by univariate Cox regression (*p* < 0.01). **(B, C)** Further selection of the optimal parameter (lambda) by LASSO Cox regression. **(D)** Establishment of the prognostic signature with 8 glycolysis-related genes based on multivariate Cox regression analysis. DLBCL, diffuse large B-cell lymphoma; LASSO, least absolute shrinkage and selection operator.

**FIGURE 2:**
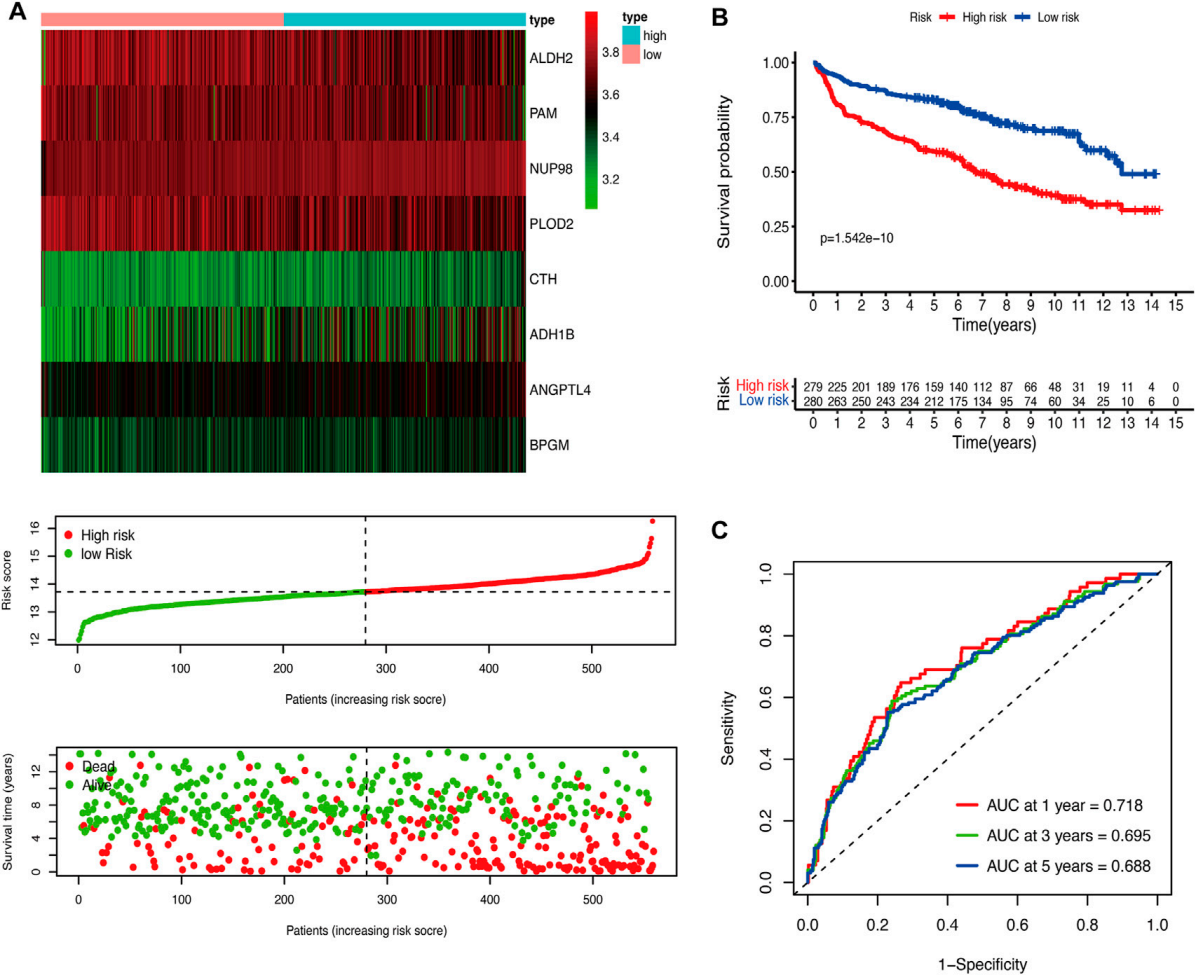
Construction of the glycolytic risk model for patients with DLBCL in the training cohort. **(A)** Glycolysis-related risk gene expression, risk score distribution, and survival status of patients in high- and low-risk groups. **(B)** Kaplan-Meier analysis of overall survival in different risk groups. **(C)** Time-dependent ROC analysis of the glycolytic risk model. DLBCL, diffuse large B-cell lymphoma; ROC, receiver operating characteristic.

We performed the univariate Cox regression analysis with the gene expression profile and survival information of DLBCL patients in the training cohort and identified 14 prognostic genes related to OS (*p* < 0.01), including eight protective genes (hazard ratio (HR) < 1.0) and six risk genes (HR > 1.0, Figure 1A). Lasso regression (Figures 1B, C) and multivariate analyses (Figure 1D) were further applied to screen prognostic genes. We established a prognostic signature composed of eight glycolysis-related genes in DLBCL, eventually. The glycolytic risk score for OS = (*ADH1B* × 0.17355739) + (*ALDH2* × −0.121597937) + (*ANGPTL4* × 0.301463871) + (*BPGM* × 0.396173449) + (*CTH* × 0.196458723) + (*NUP98* × 0.655960453) + (*PAM* × −0.14314632) + (*PLOD2* × −0.144167963). Among these prognostic genes, *ADH1B* (*p* < 0.001), *ANGPTL4* (*p* = 0.030), *BPGM* (*p* < 0.001), *NUP98* (*p* < 0.001), and *PAM* (*p* = 0.046) were independent risk factors for survival. Based on the risk score, 559 patients were assigned into high-risk (*n* = 279) and low-risk groups (*n* = 280) using the median risk score as the cutoff threshold. As revealed by the heatmap, *ADH1B*, *ANGPTL4*, *BPGM*, *CTH*, and *NUP98* were significantly up-regulated while *ALDH2*, *PAM*, and *PLOD2* were down-regulated in the high-risk group (all *p* < 0.001, Figure 2A). The risk score distribution and survival status of the individual patient indicated that the poor prognosis was tightly related to the high-risk score (Figure 2A). Patients in the high-risk group had a significantly worse survival than that in the low-risk group (*p* < 0.001, Figure 2B). The corresponding OS rates at 5-year were 57.2% and 75.7%, respectively. AUCs of the glycolytic risk model were 0.718, 0.695, and 0.688 at 1-, 3-, and 5-year, demonstrating its excellent predictive value (Figure 2C).

### 4.3 Validation of the risk model

**FIGURE 3:**
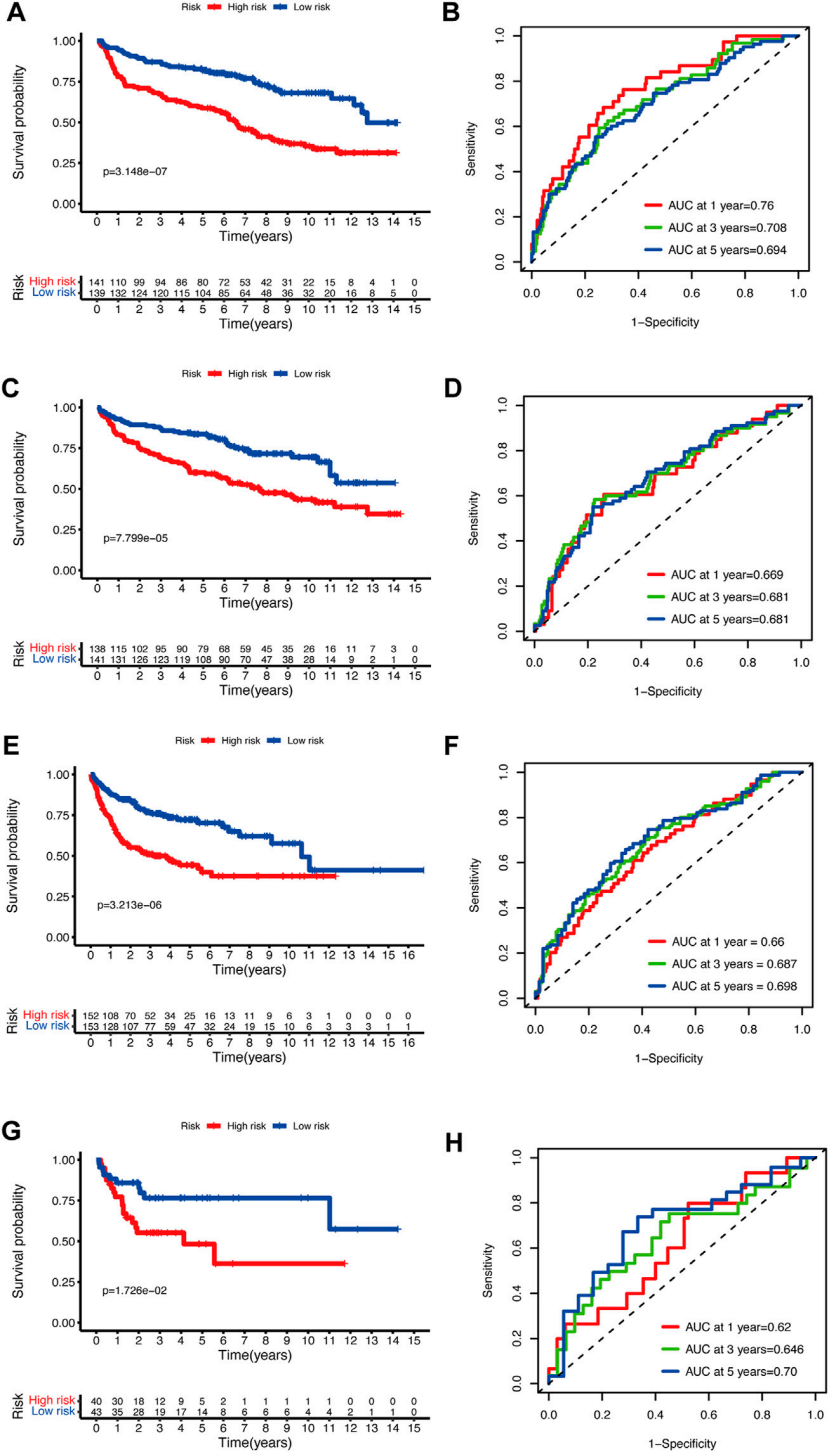
Validation of the glycolytic risk model. Kaplan-Meier analyses of overall survival for patients in high- and low-risk groups in internal validation cohorts **(A, C)**, GSE10846 **(E)**, and GSE53786 **(G)**. Time-dependent ROC analyses of the risk model in internal validation cohorts **(B, D)**, GSE10846 **(F)**, and GSE53786 **(H)**. ROC, receiver operating characteristic.

To validate the prediction performance of the glycolytic risk model in DLBCL patients, we assessed the gene signature in two external validation cohorts and two internal validation cohorts. Similarly, high-risk patients exhibited a significantly unfavorable prognosis, compared to low-risk patients (Figures 3A, C, E, G; *p* < 0.001 for internal validation cohorts and GSE10846; *p* < 0.05 for GSE53786). AUCs at 1-, 3-, 5-year ranged from 0.669 to 0.76 in two internal validation cohorts (Figures 3B, D). The range of AUC values in two external validation cohorts was between 0.62 and 0.70 (Figures 3F, H), which further confirmed the predictive efficacy of the risk model.

### 4.4 Predictive performance of the glycolytic risk model in clinical subgroups

**FIGURE 4:**
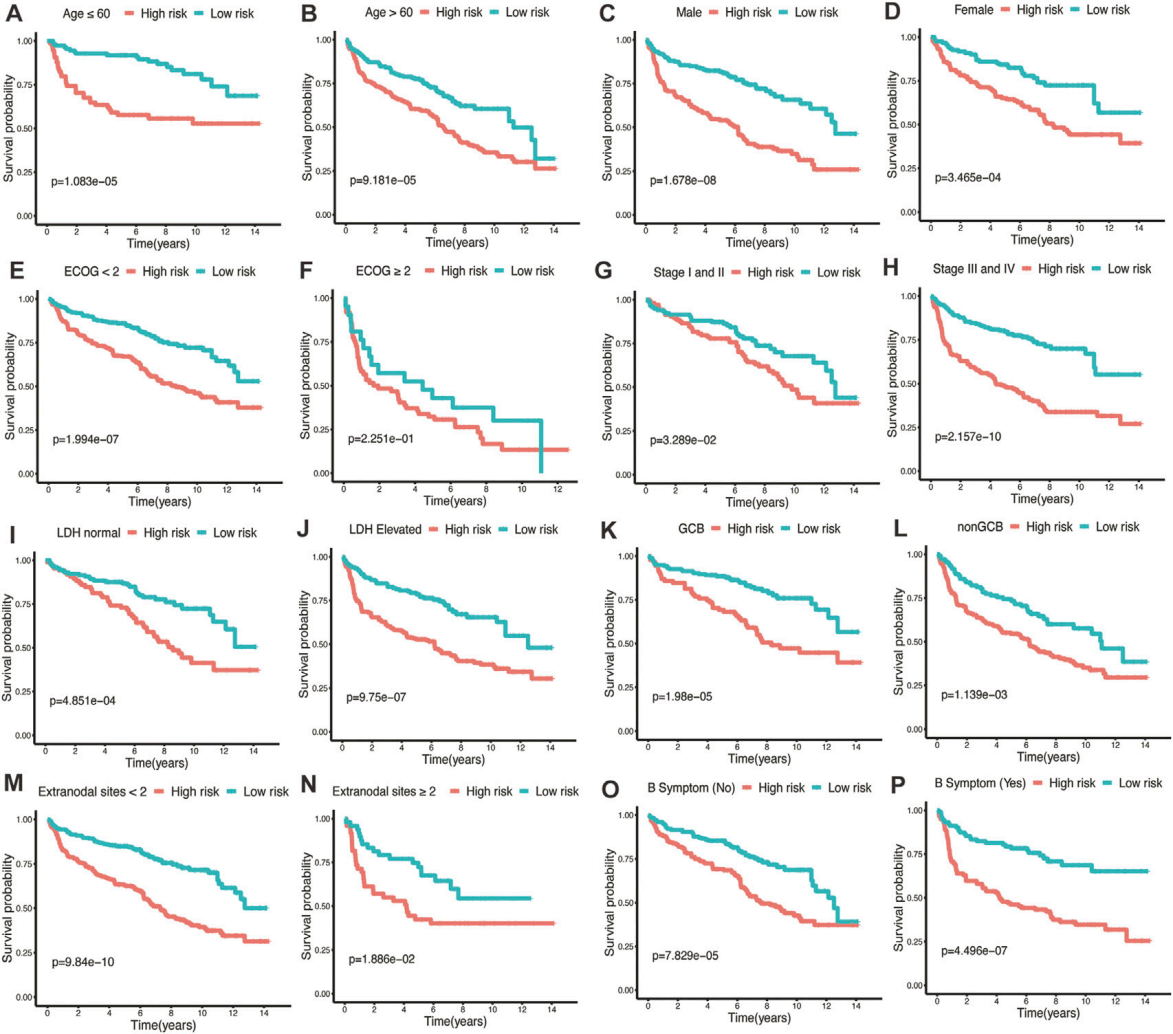
Performance of glycolytic risk model in subgroups of clinical factors. Kaplan-Meier analyses of overall survival for high- and low-risk patients in the subgroups of age **(A, B)**, gender **(C, D)**, ECOG-PS **(E, F)**, stage **(G, H)**, LDH level **(I, J)**, COO **(K, L)**, number of extranodal sites **(M, N)**, and B symptoms **(O, P)**. ECOG-PS, Eastern Cooperative Oncology Group performance status; LDH, lactate dehydrogenase; COO, cell of origin.

To assess the predictive performance of the glycolytic risk model in clinical subgroups, we conducted the clinical parameters stratification based on age (≤60 *vs*. >60), gender (male *vs*. female), ECOG-PS (<2 *vs*. ≥2), AnnAnbor Stage (I-II *vs*. III-IV), LDH (normal *vs*. elevated), COO (GCB *vs*. nonGCB), extranodal sites (<2 *vs*. ≥2), and B symptoms (no *vs*. yes) (Figures 4A–P). Overall, high-risk patients displayed poorer prognosis compared with low-risk patients regardless of age, gender, ECOG-PS, stage, LDH level, COO, number of extranodal sites, and with or without B symptoms, indicating our risk model was effective and stable.

### 4.5 Distribution of glycolytic risk scores in various clinical factors

**FIGURE 5:**
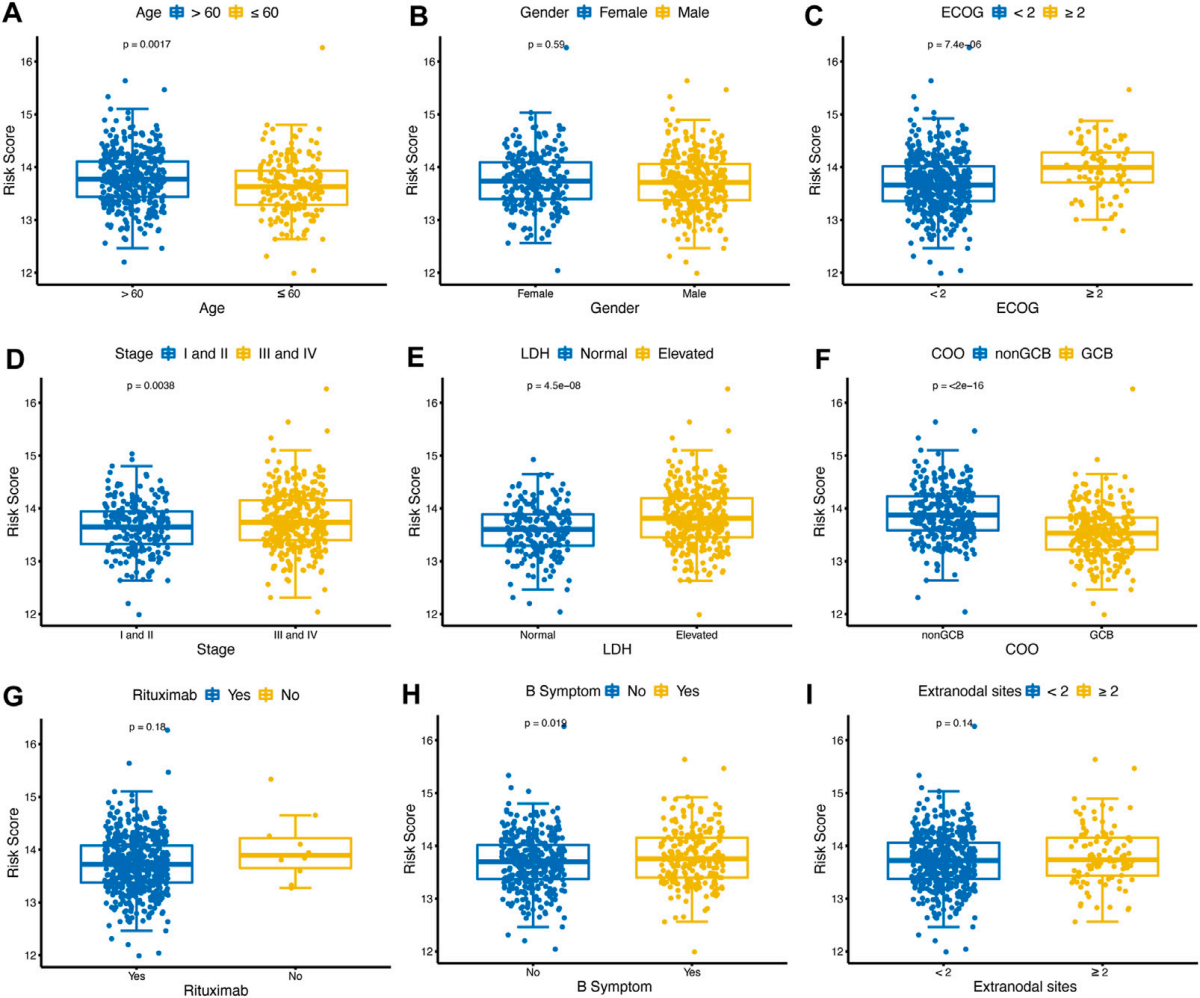
Distribution of glycolytic risk score in different clinical characteristics. Comparison of the glycolytic risk score distribution between different subgroups of age **(A)**, gender **(B)**, ECOG-PS **(C)**, stage **(D)**, LDH **(E)**, COO **(F)**, rituximab **(G)**, B symptoms **(H)**, and extranodal sites **(I)**. ECOG-PS, Eastern Cooperative Oncology Group performance status; LDH, lactate dehydrogenase; GCB, COO, cell of origin.

The distribution of risk scores was analyzed among various clinical factors in the training cohort (Figures 5A–I). Age>60 (*p* = 0.0017), ECOG-PS≥2 (*p* < 0.001), advanced stage (*p* = 0.0038), elevated LDH (*p* < 0.001), nonGCB (*p* < 0.001), and with B symptoms (*p* = 0.019) subgroups contained more high-risk patients compared with age≤60, ECOG-PS<2, early stage, normal LDH, GCB, and without B symptoms subgroups.

### 4.6 Development and validation of a glycolysis-clinical nomogram for prognostic prediction

**FIGURE 6:**
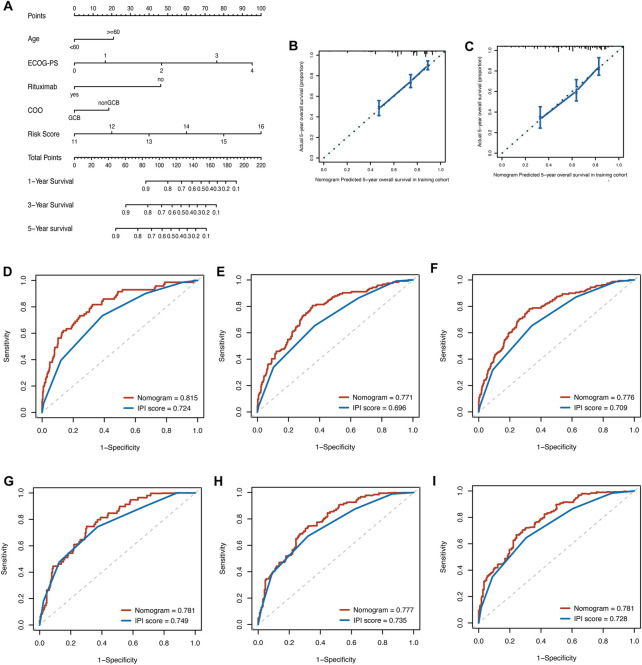
Construction of prognostic nomogram for overall survival in patients with DLBCL. **(A)** The nomogram integrated age, ECOG-PS, use of rituximab, COO, and glycolytic risk score to predict 1-, 3- and 5-year survival. **(B, C)** Calibration plots of 5-year survival probability in training cohort (GSE181063) and validation cohort (GSE10846). ROC curves of the nomogram and IPI in training cohort (GSE181063, **(D–F)** for AUCs at 1-, 3-, and 5-year, respectively) and validation cohort (GSE10846, **(G–I)** for AUCs at 1-, 3-, and 5-year, respectively). DLBCL, diffuse large B-cell lymphoma; ECOG-PS, Eastern Cooperative Oncology Group performance status; COO, cell of origin; ROC, receiver operating characteristic; IPI, international prognostic index; AUC, area under the curve.

By integrating the clinical factors and risk score, we constructed a nomogram to provide a quantitative method for predicting the 1-, 3-, and 5-year individual survival probability in DLBCL patients. Univariate Cox analysis showed that age, ECOG-PS, LDH, stage, rituximab, COO, and glycolytic risk score were OS-related factors in both training and validation cohorts (GSE10846) (Table 2, all *p* < 0.05). Multivariate Cox analysis revealed that the glycolytic risk score was an independent prognostic indicator in both cohorts (Table 2, all *p* ≤ 0.002). A higher total score in the nomogram indicated worse survival (Figure 6A). Calibration curves of the nomogram exhibited good agreement between the predicted value and the actual value (C-index = 0.73 for training cohort, Figure 6B; C- index = 0.73 for validation cohort, Figure 6C). In addition, AUCs of the nomogram at 1-, 3-, and 5-year showed its better predictive performance than IPI (Figures 6D–F for training cohort; Figures 6G–I for validation cohort), suggesting the glycolytic risk model improved the predictive value upon existing clinical predictors basis in DLBCL.

**TABLE 2:** Univariate and multivariate Cox regression analyses of clinicopathological parameters in training and validation cohorts.

Training cohort	Univariate analysis			Multivariate analysis		
	HR	95%CI	*p*-value	HR	95%CI	*p*-value
Age	2.070	1.522–2.815	<0.001	1.870	1.356–2.578	<0.001
Gender	0.777	0.601–1.003	0.053			
ECOG-PS	2.004	1.710–2.347	<0.001	1.920	1.623–2.271	<0.001
LDH	1.585	1.210–2.076	<0.001	1.043	0.782–1.393	0.774
Stage	1.267	1.123–1.428	<0.001	1.137	0.979–1.320	0.092
Rituximab	2.056	1.014–4.166	0.046	4.085	1.921–8.686	<0.001
Extranodal sites	1.424	1.035–1.959	0.030	1.084	0.744–1.582	0.674
COO	2.067	1.584–2.696	<0.001	1.622	1.225–2.148	<0.001
Risk Score	2.718	2.158–3.424	<0.001	2.117	1.607–2.789	<0.001

Validation cohort	HR		*p*-value	HR		*p*-value
Age	2.085	1.434–3.030	<0.001	1.993	1.361–2.917	<0.001
Gender	1.084	0.759–1.547	0.659			
ECOG-PS	1.760	1.472–2.103	<0.001	1.432	1.179–1.740	<0.001
LDH	2.460	1.692–3.577	<0.001	1.499	0.998–2.250	0.051
Stage	1.485	1.245–1.770	<0.001	1.325	1.096–1.601	0.004
Rituximab	1.864	1.271–2.732	0.001	1.892	1.283–2.790	0.001
Extranodal sites	1.857	0.993–3.471	0.053			
COO	2.573	1.724–3.893	<0.001	1.523	0.979–2.369	0.062
Risk Score	1.999	1.561–2.560	<0.001	1.595	1.192–2.134	0.002

HR, Hazard ratio; 95% CI, 95% Confidence interval; ECOG-PS, Eastern cooperative oncology group performance status; LDH, Lactate dehydrogenase; COO, Cell of origin.

### 4.7 Glycolytic risk model and TME

**FIGURE 7:**
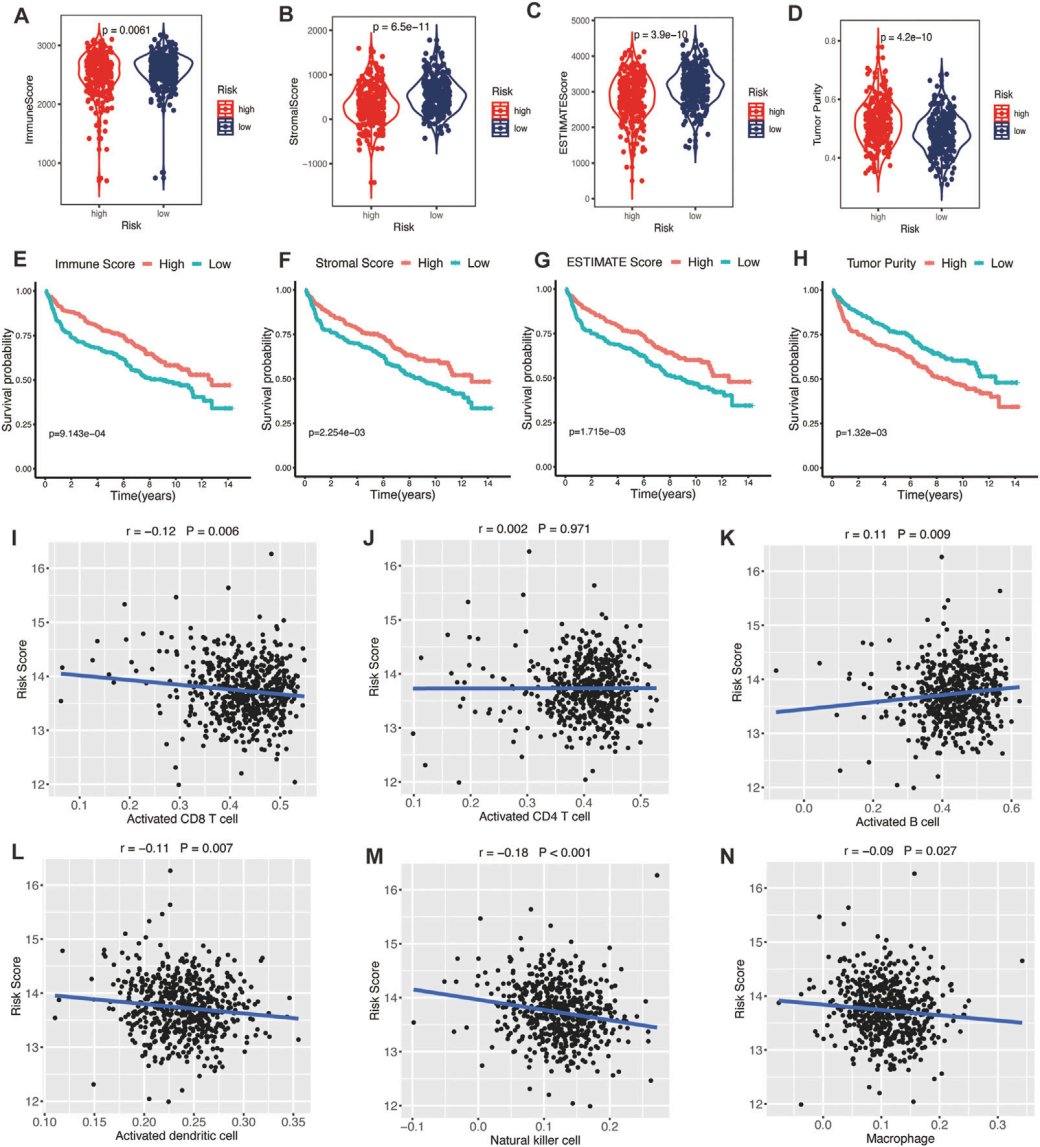
Association between glycolytic gene signature and tumor microenvironment. **(A–D)** Comparison of the immune score, stromal score, ESTIMATE score, and tumor purity between low- and high-risk patients with DLBCL. Kaplan-Meier analyses of high- and low-immune score **(E)**, stromal score **(F)**, ESTIMATE score **(G)**, and tumor purity **(H)** patients. Correlation between activated CD8 T cells **(I)**, activated CD4 T cells **(J)**, activated B cells **(K)**, activated dendritic cells **(L)**, natural killer cells **(M)**, macrophages **(N)**, and risk score. DLBCL, diffuse large B-cell lymphoma.

TME was represented by the stromal and immune scores (Liang et al., 2021). Patients in the high-risk group exhibited low immune scores, stromal scores, and ESTIMATE scores (Figures 7A–C; all *p* < 0.01) and high tumor purities (Figure 7D; *p* < 0.001), indicating the glycolytic risk model was associated with immunosuppression. The corresponding results of survival analysis (Figures 7E–G, all *p* ≤ 0.001) were coincident with the high-risk score-related poor prognosis. To explore the underlying mechanism, we analyzed the correlation between the risk model and infiltration levels of immune cells. As shown in Figure 7, the glycolytic risk score was negatively correlated with activated CD8 T cells (Figure 7I; *r* = −0.12, *p* = 0.006), activated dendritic cells (Figure 7L; *r* = −0.11, *p* = 0.007), natural killer cells (Figure 7M; *r* = −0.18, *p* < 0.001), and macrophages (Figure 7N; *r* = −0.09, *p* = 0.027), thus might explained why glycolytic risk score had a negative influence on clinical outcomes in DLBLC patients.

### 4.8 Glycolytic risk model and immune checkpoint molecules

**FIGURE 8:**
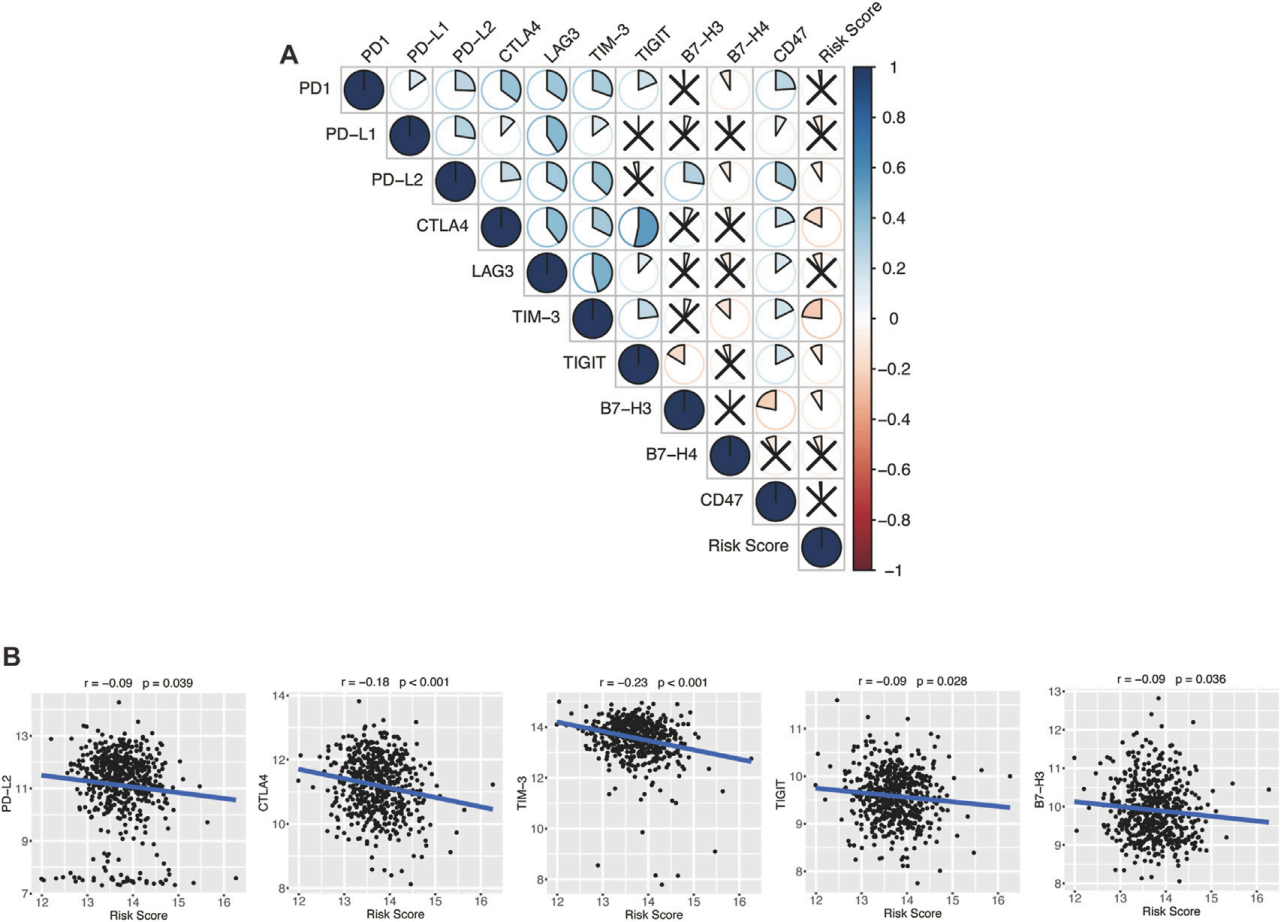
Association between the glycolytic risk model and immune checkpoint modulators. **(A, B)** Correlation between the risk score and immune checkpoint modulators. **(B)** Correlation between the risk score and expression of *PD-L2*, *CTLA4*, *TIM-3*, *TIGIT*, and *B7-H3*.

Encouraged by plenty of achievements in antitumor immunotherapy, we evaluated the correlations of our glycolytic risk model and the immune checkpoint molecules *PD-1*, *PD-L1*, *PD-L2*, *CTLA-4*, *LAG3*, *TIM-3*, *TIGIT*, *B7-H3*, *B7-H4*, and *CD47* in DLBCL. As shown in Figure 8, the glycolytic gene signature was negatively associated with *PD-L2* (*r* = −0.09, *p* = 0.039), *CTLA4* (*r* = −0.18, *p* < 0.001), *TIM-3* (*r* = −0.23, *p* < 0.001), *TIGIT* (*r* = −0.09, *p* = 0.028), *B7-H3* (*r* = −0.09, *p* = 0.036).

### 4.9 The protein expression level of risk genes

To explore the protein expression of the eight glycolytic risk genes in patients with DLBLC, we compared the protein staining intensity of these genes by using the HPA database. ADH1B, CTH, and PLOD2 showed overexpression in the lymph node tissue of non-Hodgkin lymphoma patients compared to normal lymph node tissue. Unfortunately, the information of NUP98 was not available in the HPA database. No expression difference was found of ALDH2, ANGPTL4, BPGM, and PAM between lymphoma patients and control samples (Supplementary Figure S1).

## 5 Identified differential metabolites

Based on the expression of risk/protective genes in high- and low-risk groups, we explored the associated metabolites and presented the 66 metabolites in the supplementary figure, such as lactate, tryptophan, isoleucine, sarcosine, citrate etc. (Supplementary Figure S2).

### 5.1 Screening of potential anti-cancer compounds

Based on the GDSC database, we assessed the drug therapeutic responses between high- and low-risk groups. Of note, patients in the high-risk group were more sensitive to cytarabine, methotrexate, vinblastine, gemcitabine, lenalidomide, and rapamycin, while patients in the low-risk group were more sensitive to docetaxel, bortezomib, bleomycin and LFM.A13, which provided the reference directing clinical treatment (Supplementary Figure S3).

### 5.2 Differential biological behaviors between high- and low-risk groups

GSVA analysis was performed to explore the difference in biological signaling pathways activated/inhibited between high- and low-risk groups. Our results showed significant differences in biological processes between high- and low-risk patients. Notably, MYC targets V2, MYC targets V1, E2F targets, G2M checkpoint, and unfolded protein response signaling were the top enriched signatures in the high-risk group. In comparison, low-risk patients were more relevant to the apical surface, coagulation, epithelial-mesenchymal transition, angiogenesis, and estrogen response early signaling pathways (Supplementary Figure S4).

## 6 Discussion

Accurate identification of high-risk DLBCL patients is important for prognostic prediction and decision-making regarding treatment choices. The IPI has been used to predict prognosis of patients with aggressive non-Hodgkin lymphoma since 1993 (Liu and Barta, 2019). However, IPI system only includes clinical characteristics and cannot directly reflect the biological heterogeneity of patients with DLBCL (International Non-Hodgkin’s Lymphoma Prognostic Factors, P, 1993). Moreover, there is no conclusive information for comparing the effects of glycolysis and OXPHOS in B-cell lymphoma. Through biological function experiments, the effects of AZD3965 and IACS-010759, the inhibitors of glycolysis and OXPHOS, were tested in 8 cell lines of B cell lymphoma. It was found that AZD3965 decreased the growth of lymphoma cells from 60% to 98% in 4 cell lines and inhibition of oxidative phosphorylation resulted in only 5%–45% inhibition of lymphoma cell growth in all cell lines (Noble et al., 2022). This confirmed that glycolysis regulates the proliferation of B cell lymphoma. In this study, we constructed and validated a glycolytic risk model in patients with DLBCL for the first time. The glycolytic risk model showed good prediction performance for clinical outcomes and characteristics of TIME, metabolites, activated signaling pathways, and the sensitivity of patients to chemotherapy and targeted therapies in DLBCL.

Glycolysis affects tumorigenesis and cancer development *via* interacting with the TME (Takahashi et al., 2021). Indeed, we found that DLBCL patients with high glycolytic risk scores were characterized by high tumor purities and low immune scores in this study. Sukumar et al. (2013) Showed that increased glycolytic activity downregulated CD8^+^ T cell memory and antitumor function. Previous immune cell enrichment analysis in patients with head and neck squamous cell carcinoma showed that enhanced glycolysis downregulated the infiltration level of T cells, dendritic cells, and B cells, which impaired T cell activation, tumor antigen presentation, and antibody production (Martinez-Reyes and Chandel, 2021). Several glycolysis-related prognostic models have been established to predict cancer survival and immune cell infiltration in the TIME (Yu et al., 2020; Xia et al., 2021). Yu et al. (2020) constructed a glycolysis-based risk scoring model for patients with gastric cancer. They found that the level of immune-positive cells, such as the NK cells, in the low-risk group was significantly higher compared with that of the high-risk group. Particularly, the high-risk group showed high infiltration of immunosuppressive cells (Yu et al., 2020). It was postulated that high glycolytic gene expression induced impaired immunity and immunosuppressive TIME, increased risk of tumor formation, and poor prognosis (Yu et al., 2020). In hepatocellular carcinoma, Xia et al. (2021) confirmed that the glycolysis-related model was negatively correlated with CD8 T cells and NK cells, indicating that the poor prognosis of high-risk group may be driven by infiltration of various immune cells. In accord with them, we found that the glycolytic risk score was negatively correlated to the activated CD8 T cell, natural killer cell, and activated dendritic cell. CD8^+^ T cell is the preferred immune cell type in the process of targeting cancer cells (Farhood et al., 2019). Natural killer cells recognize and kill cancer cells *via* releasing cytolytic granules. Dendritic cells capture antigens and present them to the T cells during the antitumor process (Galli et al., 2020). These findings might explain the poor prognosis observed in patients of the high-risk group in the present study. In this study, the glycolytic risk score was positively correlated with activated B cells. According to cell-of-origin, DLBCL was pathologically divided into germinal center B cell like, activated B cell like, and unclassifiable subtypes. The activated B cell like subtype exhibited a poor prognosis compared with the germinal center B cell like subtype (Li et al., 2018). The correlation between glycolytic risk score and activated B cells may be explained by the pathological features of DLBCL.

The glycolytic risk model developed in our study showed excellent prediction performance and its efficacy was verified from multiple aspects. High-risk patients had significantly worse clinical outcomes than low-risk patients in all cohorts. Besides, the developed risk model still had good performance after clinical factors stratification. High- and low-risk patients showed similar prognoses in ECOG-PS>2 group as other groups, however, without significant difference, which might be caused by the small number of cases in this group. An important finding is that some specific groups, including age>60, ECOG-PS≥2, advanced stage, elevated LDH, nonGCB, and with B symptoms, encompassed more high-risk patients. Warburg found that cancer cells preferred aerobic glycolysis to oxidative phosphorylation for glucose metabolism (DeBerardinis and Chandel, 2020). However, aerobic glycolysis is inefficient compared to oxidative phosphorylation in terms of generating adenosine 5′-triphosphate. Therefore, cancer cells competitively absorb more glucose than normal cells to meet their proliferation needs (Vander Heiden et al., 2009), and this forms the basis for clinical application of FDG-PET/CT (El-Galaly et al., 2018). It has been reported that aggressive lymphomas have higher maximum standardized uptake value than indolent lymphomas, indicating that the invasion activity of lymphoma is dependent on glucose uptake (El-Galaly et al., 2018). A study by Lee et al. (2021) showed that high-grade renal cell carcinoma had higher F-18 FDG uptake than low-grade tumors and F-18 FDG uptake was positively associated with high Fuhrman grades. These findings suggest that advanced stage and invasive disease status tend to correlate with increased glucose metabolism than early-stage disease and this might explain our results. However, further experiments are needed to verify our hypothesis.

In the past decade, ICIs has revolutionized cancer therapy (Robert, 2020). Several clinical trials have demonstrated that ICIs can effectively treat malignant lymphoma (Li et al., 2021). However, only a proportion of patients show satisfactory response to ICIs. To find effective biomarkers for predicting ICI response, Hu et al. performed a comprehensive analysis at a pan-cancer level and found that low expression of immune checkpoint gene indicated worse response to ICI immunotherapy, low immune infiltration, and poor prognosis (Hu et al., 2021). Ren et al. (2018) reported that higher PD-1 expression was correlated with favorable disease-free survival and OS in breast cancer patients. In our study, the glycolytic gene signature was negatively correlated with immune checkpoint modulator gene expression, including PD-L2, CTLA-4, TIM-3, TIGIT, and B7-H3, which might indicate that high-risk patients with DLBCL have a poor response to the immunotherapy of these targets. However, biomarkers for predicting the response to ICIs are not only based on these target molecules but also on other factors like antigen presentation, tumor mutational burden, and interferon-gamma release (Hatic et al., 2021).

In this retrospective study, we comprehensively analyzed the prognosis and immune environments of patients with DLBCL based on glycolysis. However, our findings are based on the analysis of the online databases and are short of further experimental validation. Further large-scale and prospective cohort studies are thus required to confirm our findings. Moreover, in-depth *in vivo* and *in vitro* experiments are necessary to be done to elucidate the biological function of glycolysis-related risk genes and to explore the underlying mechanism.

## 7 Conclusion

In conclusion, the glycolytic risk model and the nomogram developed in our study provided promising and stable prognostic predictors in patients with DLBCL. Our results indicated that the glycolytic risk model might provide potential therapeutic targets in clinical practice and reflected immune status, giving insights into the underlying mechanism of DLBCL.

## Data Availability

The datasets presented in this study can be found in online repositories. The names of the repository/repositories and accession number(s) can be found in the article/Supplementary Material.
