# Supplementary figures and images for: A glycolysis-related gene signatures in diffuse large B-Cell lymphoma predicts prognosis and tumor immune microenvironment
Source: Front Cell Dev Biol. 2023 Jan 23;11:1070777. doi: 10.3389/fcell.2023.1070777 (PMC9899826; doi:10.3389/fcell.2023.1070777)

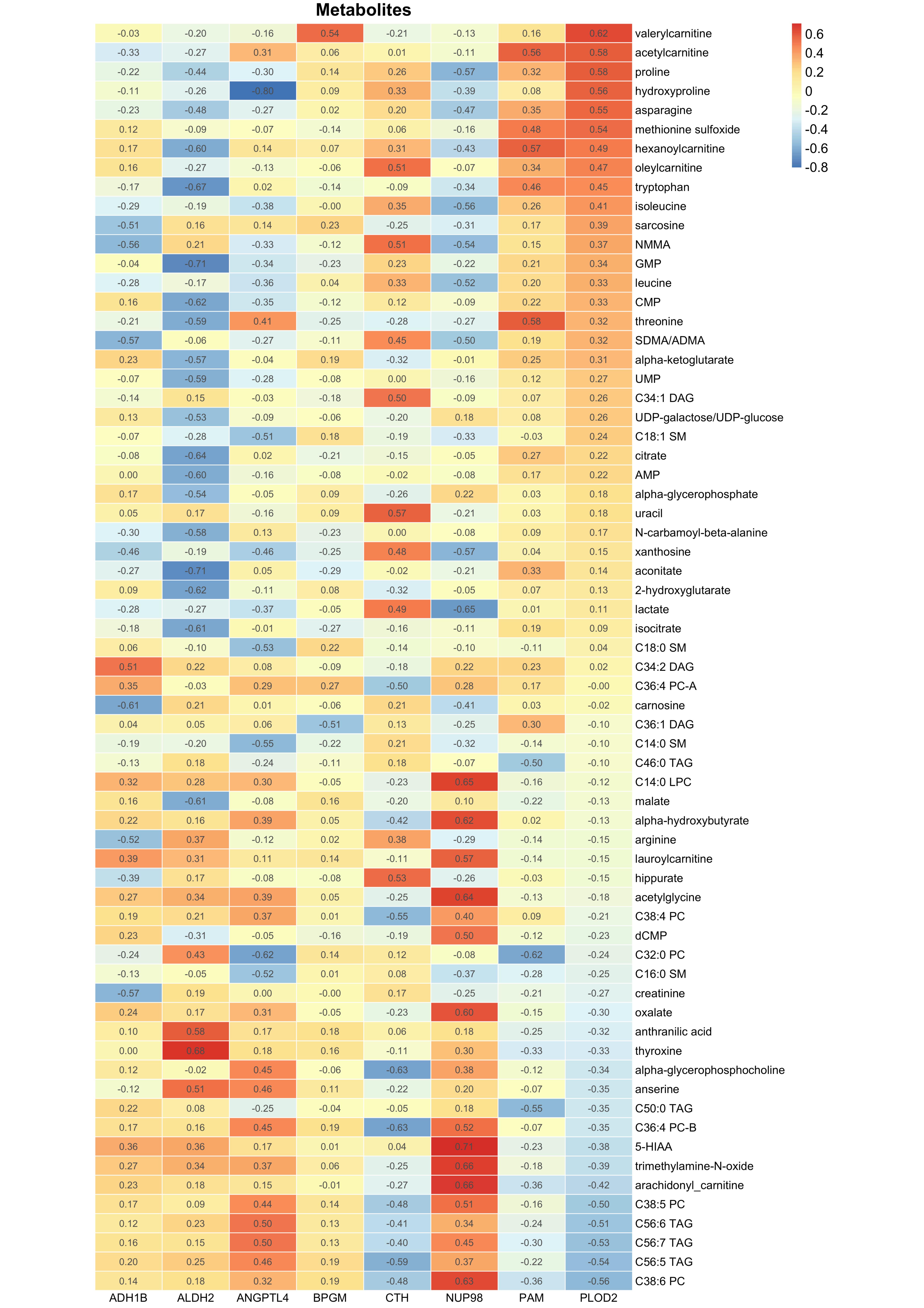

Supplement: Supplementary file 1 [file Image2.jpg]

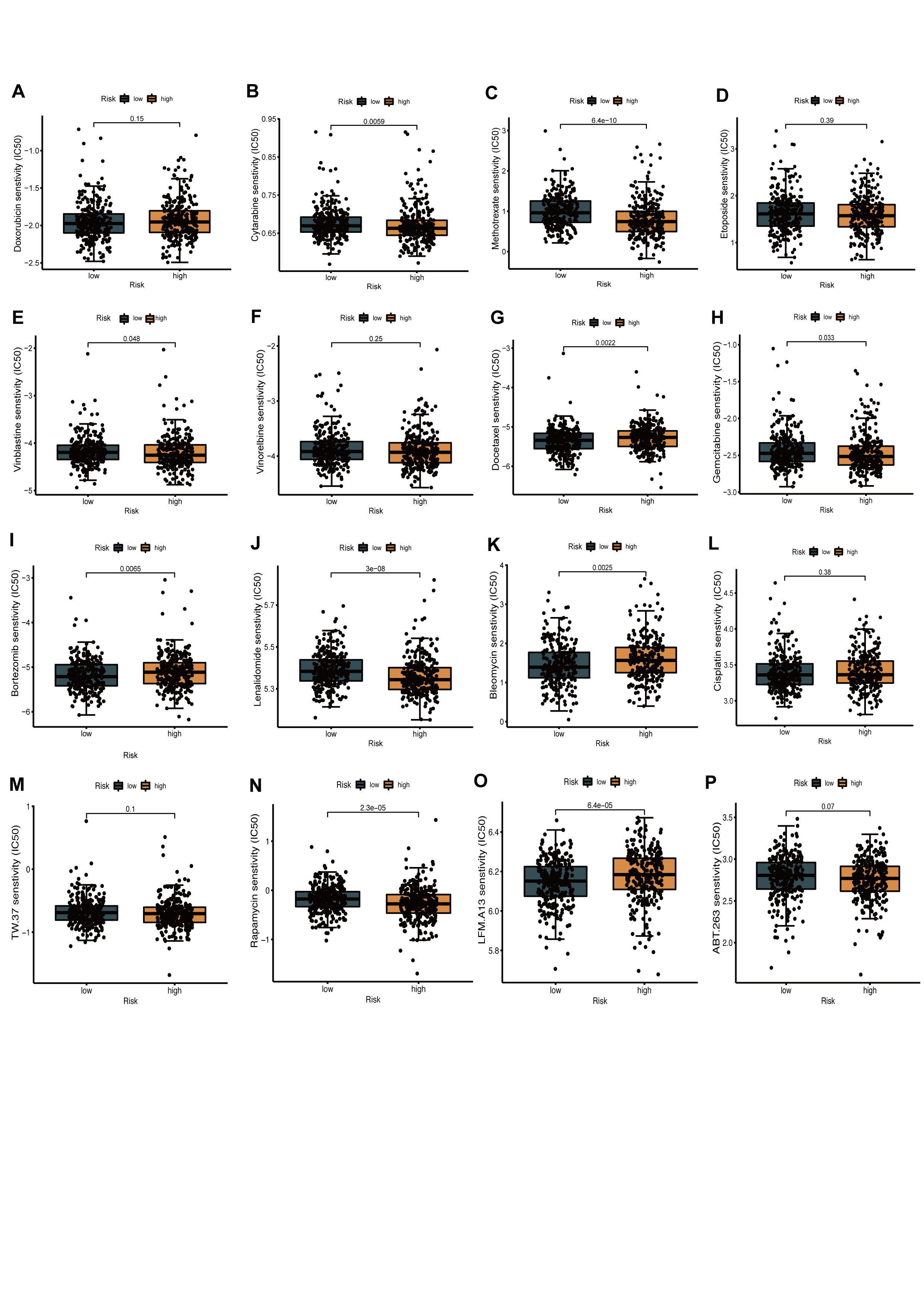

Supplement: Supplementary file 3 [file Image3.TIF]

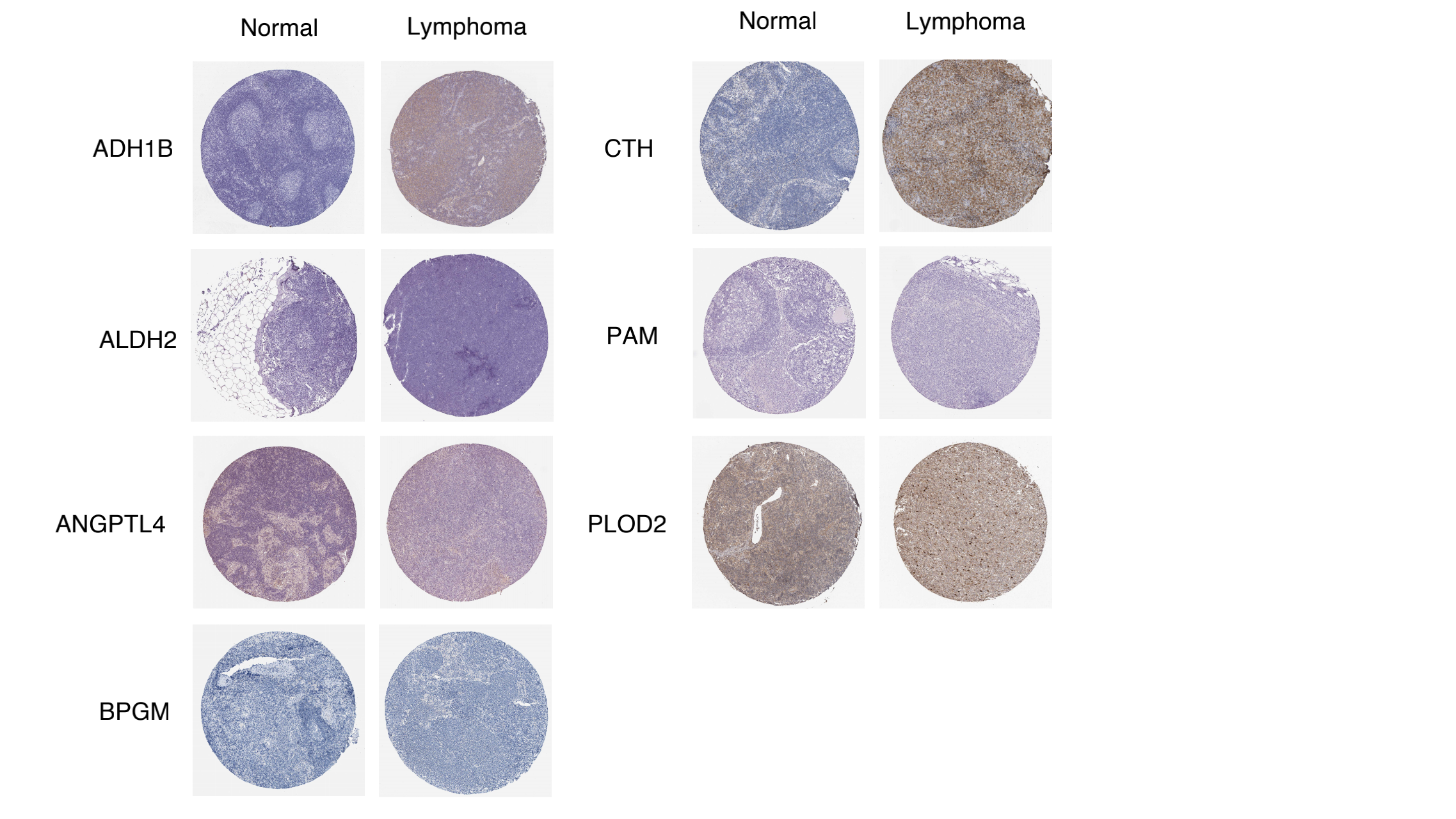

Supplement: Supplementary file 5 [file Image1.TIF]
