# Supplementary material for: A glycolysis-related gene signatures in diffuse large B-Cell lymphoma predicts prognosis and tumor immune microenvironment
Source: Front Cell Dev Biol. 2023 Jan 23;11:1070777. doi: 10.3389/fcell.2023.1070777 (PMC9899826; doi:10.3389/fcell.2023.1070777)
Supplement: Supplementary file 2 [file Table1.docx]

Supplementary Material

A Glycolysis-Related Gene Signatures in Diffuse Large B-Cell Lymphoma Predicts Prognosis and Tumor Immune Microenvironment

Yingying Cui^1,2†*^, Changsen Leng^1,3,4†*^

^1^State Key Laboratory of Oncology in South China, Collaborative Innovation Center for Cancer Medicine, Sun Yat-sen University Cancer Center, Guangzhou 510060, P. R. China.

^2^Department of Hematologic Oncology, Sun Yat-sen University Cancer Center, Guangzhou 510060, P. R. China.

^3^Department of Thoracic Surgery, Sun Yat-sen University Cancer Center, Guangzhou 510060, P. R. China

^4^Guangdong Esophageal Cancer Institute, Guangzhou 510060, P. R. China.

Correspondence to: Changsen Leng, [lengcs@sysucc.org.cn](mailto:lengcs@sysucc.org.cn) and Yingying Cui, [cuiyy@sysucc.org.cn](mailto:cuiyy@sysucc.org.cn);

^†^ These authors have contributed equally to this work and share first authorship

**Figure legend**

**Figure S1.** Comparison of the protein expression level of the glycolytic risk genes between the lymphoma patients and normal controls.

**Figure S2.** A total of 66 associated metabolites based on the expression of risk/protective genes.

**Figure S3.** Potential anti-cancer drugs in DLBCL with differential IC50 between high- and low-risk groups.

**Figure S4.** Exploration of the differences of biological processes between high- and low-risk patients with GSVA analysis.

**Table S1** clinicopathological characteristics of patients in two internal validation cohorts.

|  |  | **Internal validation  cohort 1 (N=280)** |  | **Internal validation cohort 2 (N=279)** |  | ***P* value** |  | **Overall (N=559)** |
| --- | --- | --- | --- | --- | --- | --- | --- | --- |
|  |  |  |  |  |  |  |  |  |
| Age (year), No. (%) |  |  |  |  |  |  |  |  |
| ≤60 |  | 96 (34.3%) |  | 88 (31.5%) |  | 0.548 |  | 184 (32.9%) |
| >60 |  | 184 (65.7%) |  | 191 (68.5%) |  |  |  | 375 (67.1%) |
| Gender, No. (%) |  |  |  |  |  |  |  |  |
| Male |  | 155 (55.4%) |  | 150 (53.8%) |  | 0.769 |  | 305 (54.6%) |
| Female |  | 125 (44.6%) |  | 129 (46.2%) |  |  |  | 254 (45.4%) |
| ECOG-PS, No. (%) |  |  |  |  |  |  |  |  |
| <2 |  | 239 (85.4%) |  | 237 (84.9%) |  | 1 |  | 476 (85.2%) |
| ≥2 |  | 41 (14.6%) |  | 42 (15.1%) |  |  |  | 83 (14.8%) |
| LDH, No. (%) |  |  |  |  |  |  |  |  |
| Normal |  | 106 (37.9%) |  | 108 (38.7%) |  | 0.904 |  | 214 (38.3%) |
| Elevated |  | 174 (62.1%) |  | 171 (61.3%) |  |  |  | 345 (61.7%) |
| AnnAnbor stage, No. (%) |  |  |  |  |  |  |  |  |
| I–II |  | 113 (40.4%) |  | 108 (38.7%) |  | 0.755 |  | 221 (39.5%) |
| III–IV |  | 167 (59.6%) |  | 171 (61.3%) |  |  |  | 338 (60.5%) |
| COO, No. (%) |  |  |  |  |  |  |  |  |
| GCB |  | 133 (47.5%) |  | 132 (47.3%) |  | 1 |  | 265 (47.4%) |
| nonGCB |  | 147 (52.5%) |  | 147 (52.7%) |  |  |  | 294 (52.6%) |
| Rituximab, No. (%) |  |  |  |  |  |  |  |  |
| Yes |  | 274 (97.9%) |  | 275 (98.6%) |  | 0.754 |  | 549 (98.2%) |
| No |  | 6 (2.1%) |  | 4 (1.4%) |  |  |  | 10 (1.8%) |
| B Symptom, No. (%) |  |  |  |  |  |  |  |  |
| No |  | 160 (57.1%) |  | 178 (63.8%) |  | 0.128 |  | 338 (60.5%) |
| Yes |  | 120 (42.9%) |  | 101 (36.2%) |  |  |  | 221 (39.5%) |
| Extranodal sites, No. (%) |  |  |  |  |  |  |  |  |
| <2 |  | 233 (83.2%) |  | 229 (82.1%) |  | 0.808 |  | 462 (82.6%) |
| ≥2 |  | 47 (16.8%) |  | 50 (17.9%) |  |  |  | 97 (17.4%) |

No., number; DLBCL, diffuse large B-cell lymphoma; ECOG-PS, Eastern Cooperative Oncology Group performance status; LDH, lactate dehydrogenase; COO, cell of origin; GCB, germinal center B cell; IPI, international prognostic index.
